# Reliability and feasibility of gait initiation centre-of-pressure excursions using a Wii^®^ Balance Board in older adults at risk of falling

**DOI:** 10.1007/s40520-018-0945-6

**Published:** 2018-04-17

**Authors:** James Lee, Graham Webb, Adam P. Shortland, Rebecca Edwards, Charlotte Wilce, Gareth D. Jones

**Affiliations:** 1 Faculty of Life Sciences and Medicine, Academic Department of Physiotherapy, Guy’s Campus, King’s College London, London, SE1 1UL UK; 2 One Small Step Gait Laboratory, Guy’s Hospital, Guy’s & St Thomas’ Foundation NHS Trust, London, SE1 7EH UK; 3 Division of Imaging Science and Biomedical Engineering, King’s College, London, SE1 9RT UK; 4 Physiotherapy Department, 3rd Floor Lambeth Wing, St Thomas’ Hospital, Westminster Bridge Road, Guy’s & St Thomas’ NHS Foundation Trust, London, SE1 7EH UK; 5 Faculty of Life Sciences & Medicine, Centre for Human & Applied Physiological Sciences (CHAPS), Guy’s Campus, King’s College London, London, SE1 1UL UK

**Keywords:** Gait initiation, Balance function, Rehabilitation, Falls, Reliability, Wii balance board

## Abstract

**Background:**

Impairments in dynamic balance have a detrimental effect in older adults at risk of falls (OARF). Gait initiation (GI) is a challenging transitional movement. Centre of pressure (COP) excursions using force plates have been used to measure GI performance. The Nintendo Wii Balance Board (WBB) offers an alternative to a standard force plate for the measurement of CoP excursion.

**Aims:**

To determine the reliability of COP excursions using the WBB, and its feasibility within a 4-week strength and balance intervention (SBI) treating OARF.

**Methods:**

Ten OARF subjects attending SBI and ten young healthy adults, each performed three GI trials after 10 s of quiet stance from a standardised foot position (shoulder width) before walking forward 3 m to pick up an object. Averaged COP mediolateral (ML) and anteroposterior (AP) excursions (distance) and path-length time (GI-onset to first toe-off) were analysed.

**Results:**

WBB ML (0.866) and AP COP excursion (0.895) reliability (ICC_3,1_) was excellent, and COP path-length reliability was fair (0.517). Compared to OARF, healthy subjects presented with larger COP excursion in both directions and shorter COP path length. OARF subjects meaningfully improved their timed-up-and-go and ML COP excursion between weeks 1–4, while AP COP excursions, path length, and confidence-in-balance remained stable.

**Discussion:**

COP path length and excursion directions probably measure different GI postural control attributes. Limitations in WBB accuracy and precision in transition tasks needs to be established before it can be used clinically to measure postural aspects of GI viably.

**Conclusions:**

The WBB could provide valuable clinical evaluation of balance function in OARF.

## Introduction

There is a significant association between ageing and fall prevalence [1, 2] with concomitant economic impact costing the UK National Health Service (NHS) £2 billion per year [3]. Despite this, risk factors for falls such as lack of flexibility, muscle weakness, poor mobility and balance confidence are modifiable if systematic exercise-based interventions are employed [1, 2]. These are often physiotherapist-led prevention programmes for older adults who have fallen or are at risk of falling [4]. Typically, they include strength and balance (S + B) exercises monitored by reliable and valid clinical measures of balance and mobility limitations [5]. However, these clinical measures are limited by ceiling effects and the inability to detect small changes [6].

Gait initiation (GI), an important movement in day-to-day function, represents the transition from stable bipedal stance to unstable locomotion [7, 8]. GI confers risk of falling by its requirement to transfer body weight before movement has begun [9]. Here, the typical postural phase of GI movement involves the net centre of pressure (COP_net_) displacing laterally (COP_X_) and posteriorly (COP_y_) towards the swing-limb before rapidly moving towards the stance limb as swing-limb movement begins. This COP_net_ displacement acts to uncouple it from the whole-body-centre-of-mass (BCOM), resulting in forward momentum necessary for GI and forward walking to occur [10]. Compared to healthy individuals, shorter posterolateral excursion of the COP_net_ during GI has been observed with pathology and is associated with reduced positional stability [7, 8, 11]. It is, therefore, conceivable that COP_net_ excursion during GI could provide a complementary approach in evaluating positional stability changes in addition to existing clinical measures.

However, COP_net_ excursion studies have used expensive force plates (FPs) in laboratories which are rare in clinical environments. One realistic method where COP_net_ changes could be observed during GI in clinical practice is using a Nintendo^®^ Wii Balance Board (WBB) (Nintendo®, Kyoto, Japan). One previous study testing standing balance compared WBB with a FP (AMTI Model OR6-5, Watertown, MA, USA) and demonstrated favourable reliability and validity although minimal detectable changes in COP_net_ path length were larger using the WBB [12]. Another study which used 12 WBBs found low inter-device variability in COP_net_ measurements. However, the same study also observed COP_net_ errors when compared to a laboratory-grade FP that were positively correlated with sway and amplitude of the source signal, with calibrations necessary and successfully deployed to reduce the errors [13].

Whilst there is optimism in the clinical community that the WBB can offer an affordable measurement solution, it nonetheless remains unknown if the WBB is a reliable way with which to measure positional stability during GI. Furthermore, no study to date has utilised the WBB when assessing COP_net_ changes throughout GI in an older clinical population at risk of falls. Therefore, the first aim of this study was to determine whether the use of the WBB can provide reliable COP_net_ excursion data during GI in a sample of healthy subjects. The second aim was to assess the feasibility of the WBB within an existing clinical falls environment and determine its clinical utility as a discriminatory measure within a pragmatic sample of older adults at risk of falling (OARF).

## Methods

### Design and ethics

A cross-sectional observational study was undertaken to establish the test–retest reliability of COP_net_ excursion measurements during GI in healthy volunteers, and changes in COP excursion pre-post S + B intervention (SBI) in OARF patients. The study did not receive any specific grant from funding agencies in the public, commercial, or not-for-profit sectors. It received ethical approval from the Guy’s and St Thomas’ NHS Foundation Trust Therapies Directorate Governance Committee (project no: 12234), which provides oversight for human ethics in compliance with the Helsinki Declaration.

### Subjects

Healthy subjects consisted of local university students who received prior information regarding the study and whom thereafter provided informed consent to participate. Exclusion criteria were pathology over the previous calendar year affecting normal gait, lower-limb prosthetic use, and current day-to-day neuromusculoskeletal pain or dysfunction. As part of normal clinical governance arrangements, OARF patients are informed, offered, and provide implicit consent to receive SBI as an outpatient for fall management (Functional Gait Assessment Score < 15/30 [14]). During a 6-week period in July 2016, all OARF patients receiving SBI were eligible to participate as GI measurements were included within the normal intervention delivery. Patients were excluded if they were unable to walk unaided 10 m indoors, stand unassisted for 10 s, or if their mass ≥ 150 kg.

### Study protocol

Healthy subjects were invited to attend the hospital twice, 1 week apart and undertake the GI measurement protocol (see below). OARF subjects attended the S + B clinic as per normal clinical practice. After a warm up of sitting-to-standing and major joint stretches, their intervention consisted of seven, 90 s circuit-based activities targeting lower-limb strength, static and dynamic balance, with vestibular system emphasis as appropriate. All OARF subjects were tested at weeks 1 and 4 in addition to their routine clinical tests (TUAG, gait velocity (4 m walk time), ConfBal (scores range from 10 [confident] to 30 [no confidence]) [15–17]).

#### GI measurement protocol

**Fig. 1:**
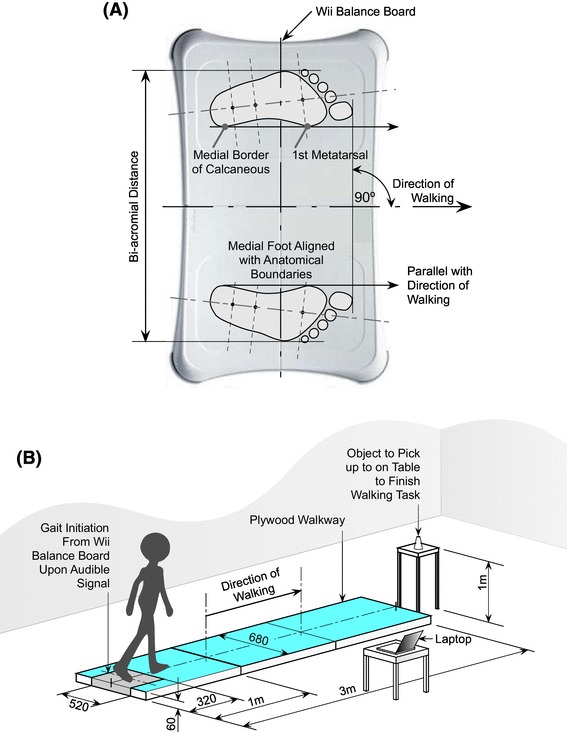
Feet starting position and walkway set up for GI COP measurement trial. **a** Subjects wearing non-slip socks adopted quiet standing for at least 10 s with feet orientated on WBB at bi-acromial (shoulder) width, with medial borders in line with direction of walking. **b** Schematic shows three plywood interconnected sections; the first section accommodating the WBB. On an audible “go” signal, standing subjects walked forward at a self-selected tempo approximately 3 m stopping to pick up an object set in midline on a table. The investigator commenced and ceased 2-dimensional recording of COP_net_ position at 60 Hz by operating a laptop wirelessly connected to the WBB, yielding COP position data from the beginning of quiet standing to the end of the second toe-off per trial. Dimensions in mm unless otherwise stated (not to scale)

A single investigator measured GI, with another person present to manage subject risk. After determining subject height unshod (Seca 213 Portable Measuring Rod, Seca Corporation, Hanover, MD), weight (seca 956 Digital Sit On Scales, Seca, Hamburg, Germany), and limb dominance (by verbal answer to the question: “Which hand do you normally write with?”), subjects were asked to don non-slip socks (Fall Prevention Slippers; Medline Industries Inc, Mundelein, IL). Bi-acromial (shoulder) width was measured (Vernier calipers; Chicago Brand, Medford, OR) to enable standardised initial foot placement on the WBB (Fig. 1a). After familiarisation, subjects were instructed to adopt a quiet-stance position for 10 s [using a handheld stopwatch (Quantum 536; Saturn Sports Ltd, March, Cambs, UK)] on the WBB before initiating walking forwards upon an audible “go” signal. Subjects were asked to walk 3 m at self-selected speed, leading with their non-dominant limb, stop, and pick up a cone with their dominant hand (Fig. 1b). Subjects undertook three trials at each visit.

The investigator commenced and ceased measurement using a locally designed graphical user interface (GUI) on a laptop computer developed using commercially available software (LabVIEW, National Instruments; Austin, TX). Horizontal 2-dimensional coordinate COP_net_ data collected at 60 Hz were transferred and stored on the laptop via wireless connectivity. The GUI required calibration at each measurement session which took approximately 10 min.

### Measurements

**Fig. 2:**
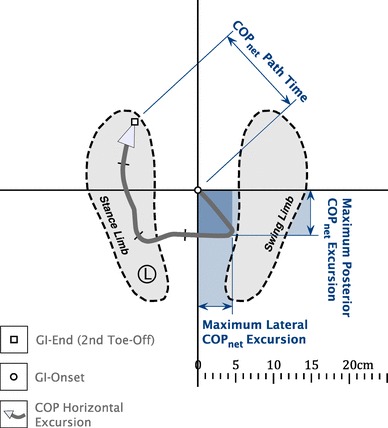
Primary COP_net_ measures. Schematic plan view of GI showing right foot as swinging limb with approximate COP_net_ path shown. Three primary measures are outlined

COP_net_ excursion data were reduced to three parameters (Fig. 2); (1) mean maximum mediolateral (ML) horizontal distance (COP_x_), (2) mean maximum anteroposterior (AP) horizontal distance (COP_y_), and (3) the COP_net_ path time (GI-onset to second toe-off time).

To establish initial COP_net_ position prior to GI, the mean (SD) position of the ML (COP_x_) and AP (COP_y_) coordinates during 10 s of quiet stance were calculated per trial enabling starting position coordinates in the WBB reference frame to be established. They were then used as offsets to translate all COP_net_ data to a standardised origin (0, 0). The instance (frame) of GI-onset was determined when COP_y_ displaced posteriorly for > 8 frames (133 ms) beyond the mean-3SD position during the first 10 s of quiet stance. The COP path time could then be calculated by subtracting the time frame at GI onset from the final COP trajectory time frame (instant of 2nd toe-off). All data were averaged across three trials for analysis, with COP_net_ displacement data normalised as a percentage of subject stature [18].

### Statistical analyses

Normality of data was confirmed (Shapiro–Wilk 1-sample test, PASW v18.0, IBM Corp., Armonk, NY). Intraclass correlation coefficients (ICC_3,1_) for absolute agreement were used to determine the test–retest COP_net_ reliability using an accepted classification system [19]. The change in normalised maximum COP_net_ excursions and path time between weeks 1–4 was analysed using paired sample Student’s *t* tests. Statistical testing was undertaken using SPSS Statistics v23 (IBM Corp., Armonk, NY) with statistical significance assumed when *p* ≤ 0.05.

## Results

Ten healthy subjects (mean±SD; 5F, 5M 26.2 ± 2.9 years, 173.9 ± 8.8 cm, 73.3±11.9 kg) and ten OARF patients attending the SBI class (8F, 2M: 83.5 ± 10.4 years, 157.1 ± 8.7 cm, 72.8 ± 14.6 kg) participated. Mean bi-acromial (shoulder) healthy and older patient width was respectively 388 ± 32 and 357 ± 32 mm. One healthy and two OARF subjects were left-limb dominant. No untoward clinical events occurred or were reported during the testing procedure. The normal SBI dose was delivered to all OARF patients.

Healthy subjects demonstrated significantly greater mean maximum COP_net_ excursions using the WBB during GI compared to OARF patients laterally [COP_x_*t*(18) = 4.619, *p* < 0.0005] and posteriorly [COP_y_*t*(18) = 6.325, *p* = < 0.0005]. Furthermore, healthy subject COP path length was shorter in duration than OARF patients which was also statistically significant [*t*(18) = 3.915, *p* = 0.003] (Table 1).

**Table 1:** Mean (± SD) max COP_net_ excursions and COP path time

Variable	Patient group	Healthy group		
Max ML (COP_x_)				
(mm)	40.62 (± 12.11)	71.35 (± 17.21)		–
(% stature)	2.07 (± 1.83)	4.11 (± 0.98)		***
Max AP (COP_y_)				
(mm)	23.50 (± 6.92)	60.16 (± 16.98)		–
(% stature)	1.49 (± 0.40)	3.50 (± 0.99)		***
COP_net_ path time				
(s)	5.45 (± 3.06)	1.65 (± 0.21)		**

Comparisons between patient and healthy groups are shown with both distance measurement (in mm) and standardised to % stature

*COP* centre of pressure, *M/L* mediolateral direction, *A/P* anteroposterior direction

*Statistically significant at *p* < 0.05, **p<0.005, ***p < 0.0005

COP_net_ within-healthy subject reliability was excellent both laterally (COP_x_) and posteriorly (COP_y_) with COP_net_ path-length time yielding fair reliability (Table 2).

**Table 2:** Within-subject reliability results

Dependent variable	ICC_3,1_	95% CI
COP_x_ (mm)	0.895	(0.676–0.988)
COP_y_ (mm)	0.866	(0.586–0.984)
COP_net_ path time (s)	0.517	(0.490–0.942)

Significant statistical improvement was observed in mean (± SD) TUAG between SBI weeks 1 (24.1 s ± 9.3) and 4 (18.2 s ± 6.5) [*p* = 0.006]. However, there was no statistically significant change (week 1–week 4) in 4 m gait time (8.5 s ± 3.4–7.8 s ± 2.5) or confBAL (19.0/30 ± 3.2–18.5/30 ± 2.8).

**Fig. 3:**
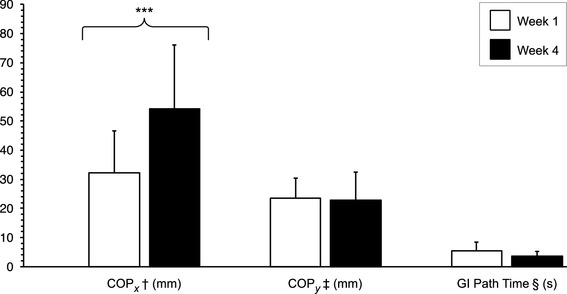
Mean (± SD) patient group WBB measures. Comparisons between weeks 1 and 4 of the S + B interventions are shown. ^†^Centre of pressure lateral excursion, ^‡^centre of pressure posterior excursion, ^§^centre of pressure path time, ***statistically significant difference at *p* < 0.00

While significant improvements in COP_x_ maximal excursion [95% CI 7.731–19.521, *p* = 0.001] were observed, neither significant change in COP_y_ maximal excursion nor COP_net_ path-length time was found between weeks 1–4 (Fig. 3).

## Discussion

The main findings of this study are that the Nintendo^®^ Wii Balance Board has excellent reliability in assessing COP excursions in both lateral and posterior directions and fair reliability in assessing COP path-length time during GI in healthy subjects. Its portability and low cost means it is attractive as a clinical measurement device. It was well tolerated by vulnerable OARF patients and was successfully incorporated into an existing S + B intervention with no untoward events observed.

Previous studies with older community-dwelling subjects have confirmed excellent reliability of COP path excursion and velocity in stance tasks using a FP [20]. A finding that has been repeated with young healthy subjects in standing using a FP and a WBB [12], and with stroke individuals using a WBB [21]. Our excellent reliability of COP excursions in GI using a WBB adds to the evidence supporting WBB use in clinical practice.

We observed that mean ML and AP COP excursions during GI in OARF patients were reduced (40.6 mm and 23.5 m) compared to healthy individuals (71.4 and 60.2 mm). This was not unexpected because reduced excursion magnitudes with advancing age or disability have been reported previously and are associated with reduced positional stability [8, 22, 23]. However, while the WBB was able to discriminate between healthy and OARF subjects, within both groups we observed larger ML compared to AP excursions which is in contrast to previous healthy individual FP data, where smaller mean (± SD) ML excursions compared to AP were reported (36.3 mm ± 0.09 and 47.0 mm ± 0.15, respectively [8]).

Two explanations are possible for this finding. First, we deployed a starting position with feet positioned shoulder-width apart with arms unconstrained. The starting position causes an unusually wide initial stance [24], but one commonly adopted in patients [25] which is why it was selected as part of a standardised protocol selected for pathological populations to tolerate safely. Nevertheless, larger ML COP excursions in GI were typical when the protocol has been adopted previously [26].

Second, while WBBs are similar to FPs in utilising four sensors near each corner, expense/quality trade-offs mean WBBs do not measure force and moments tri-axially as FPs do. Instead, WBBs measure vertical force only. Subsequently, higher magnitude ML COP position errors in WBBs compared to FPs have been reported in comprehensive validation tests [13] casting doubt about ML COP position accuracy using WBBs. This means it is possible that GI ML COP excursion using a WBB are overestimated, casting doubt on its immediate introduction to clinical practice.

In contrast to COP excursions, our healthy individual mean COP path time (1.65 s ± 0.21) was comparable to previous FP observations, where times ranged between 1.50 and 1.6 s [8, 11] and could be a more reliable parameter for the WBB’s clinical use rather than COP excursions. Our OARF mean COP path time (5.45 s ± 3.06) was substantially longer compared to previous studies (1.66–1.74 s) [8, 11]. This might be accounted for by our sample being respectively 9 and 32 years older on average, and because our group presented with a higher acuity level including a history and fear of falling. The majority of Parkinson’s Disease (PD) subjects used in previous GI literature were observed to have modified Hoehn and Yahr [27] scores of between 2.5 and 3, suggestive of moderate postural instability. While we did not measure acuity in our group, it did include patients with PD and other co-morbidities. It is plausible that our OARF group included individuals with more severe postural instability. Indeed, it was observed during our data collection that some patients found GI challenging, making several attempts before final execution which could easily have affected our overall results. We would recommend, therefore, that further WBB testing of GI COP excursions and path time include patients able to safely undertake GI unaided, but who represent as wide a spectrum of acuity and performance as is practicable.

In contrast to our reservations about immediate translation of the WBB into clinical practice now, other authors have been positive about the WBB’s measurement potential. Huurnink and colleagues concluded that WBB static balance measurements taken (mean COP sway and path velocity) were similar to those taken by a FP (mean RMS 0.31–0.74 mm; mean *r* 0.997–0.999) [28]. Yet reports of the WBB producing increased levels of noise in comparison to FP data, overestimating both COP path velocity and COP ML excursions by 3.5–5.3% [28], have added conditional exceptions on its widespread adoption. Therefore, whilst our results are positive, an experiment testing COP measurement during GI with a WBB and FP concurrently is warranted to explore specific expected errors in the WBB in this important movement task. Additionally, determining whether calibration routines can manage those errors for clinical application would be an appropriate goal of further testing.

Similar to routine clinical measures, evaluative GI function measures using a WBB during 4 weeks of SBI in the OARF group yielded mixed results within the context of error concerns mentioned above and a modest sample size. Although COP path time was quicker at week 4, the difference did not reach statistical significance. Contrastingly, maximum ML COP excursion improved significantly (increased) suggesting that a WBB is sensitive to change during the common transitional task of GI,. In the same way, clinical improvement was found in TUAG—an extensive transitional movement [29]. However, AP (backward) COP excursion did not change. Backward disequilibrium is common in older-adult postural dysfunction [30], therefore, unchanging AP COP excursions could be associated with unchanging balance confidence and its translation into gait; evidenced by non-significant changes in confBAL and gait velocity, respectively. It is possible, therefore, that COP excursions and path-time data during GI might be reflective of different aspects of balance and postural control, and warrants further investigation.

## Conclusion

We have successfully utilised the WBB to measure postural GI parameters, and yielded favourable reliability in healthy subjects. It was feasible and safe to use in an existing clinical environment for OARF. While discrimination between healthy young adults and OARF was possible, further work is required to determine WBB errors in comparison to a FP and determine the clinically meaningful differences the WBB can realistically account for. Overall, limitations in the accuracy and precision of the WBB in dynamic transition tasks need to be established before it can be used in clinical practice with confidence.

### Data availability

The datasets generated during and/or analysed during the current study are available from the corresponding author on reasonable request.
